# Antimicrobial Ionic Liquids: Ante-Mortem Mechanisms of Pathogenic EPEC and MRSA Examined by FTIR Spectroscopy

**DOI:** 10.3390/ijms25094705

**Published:** 2024-04-26

**Authors:** Patrick Mikuni-Mester, Christian Robben, Anna K. Witte, Kristina Linke, Monika Ehling-Schulz, Peter Rossmanith, Tom Grunert

**Affiliations:** 1 Centre for Food Science and Veterinary Public Health, Unit of Food Microbiology, Clinical Department for Farm Animals and Food System Science, University of Veterinary Medicine, Veterinaerplatz 1, 1210 Vienna, Austria; peter.rossmanith@vetmeduni.ac.at; 2 FFoQSI—Austrian Competence Centre for Feed and Food Quality, Safety & Innovation, Technopark 1D, 3430 Tulln, Austria; 3 Christian Doppler Laboratory for Monitoring of Microbial Contaminants, University of Veterinary Medicine, Veterinaerplatz 1, 1210 Vienna, Austria; christianrobben07@gmail.com; 4 HTK Hygiene Technologie Kompetenzzentrum GmbH, Buger Str. 80, 96049 Bamberg, Germany; anna-witte@gmx.net; 5 ZuchtData EDV-Dienstleistungen GmbH, Dresdner Str. 89/18, 1200 Vienna, Austria; linke@zuchtdata.at; 6 Centre of Pathobiology, Functional Microbiology Division, Department of Biological Sciences and Pathobiology, University of Veterinary Medicine, Veterinaerplatz 1, 1210 Vienna, Austria; monika.ehling-schulz@vetmeduni.ac.at (M.E.-S.); tom.grunert@vetmeduni.ac.at (T.G.)

**Keywords:** ionic liquids, antimicrobials, *Escherichia coli*, *Staphylococcus aureus*, FTIR

## Abstract

Ionic liquids (ILs) have gained considerable attention due to their versatile and designable properties. ILs show great potential as antibacterial agents, but understanding the mechanism of attack on bacterial cells is essential to ensure the optimal design of IL-based biocides. The final aim is to achieve maximum efficacy while minimising toxicity and preventing resistance development in target organisms. In this study, we examined a dose–response analysis of ILs’ antimicrobial activity against two pathogenic bacteria with different Gram types in terms of molecular responses on a cellular level using Fourier-transform infrared (FTIR) spectroscopy. In total, 18 ILs with different antimicrobial active motifs were evaluated on the Gram-negative enteropathogenic *Escherichia coli* (EPEC) and Gram-positive methicillin-resistant *Staphylococcus aureus* (MRSA). The results showed that most ILs impact bacterial proteins with increasing concentration but have a minimal effect on cellular membranes. Dose–response spectral analysis revealed a distinct ante-mortem response against certain ILs for MRSA but not for EPEC. We found that at sub-lethal concentrations, MRSA actively changed their membrane composition to counteract the damaging effect induced by the ILs. This suggests a new adaptive mechanism of Gram-positive bacteria against ILs and demonstrates the need for a better understanding before using such substances as novel antimicrobials.

## 1. Introduction

Since their first introduction as novel “green” solvents and enhanced reaction media, ionic liquids (ILs) have found their way into many different applications covering the entire spectrum of the natural sciences. The increasing number of IL applications and their industrialisation, particularly in organic synthesis, catalysis, extraction processes, electrochemistry and active pharmaceutical ingredients [1,2,3,4,5,6,7], has prompted investigation of their hazard potential in different biological test systems. Their environmental fate has been studied in terms of persistence in air, water and soil, as well as (bio)degradation and bioaccumulation in aquatic or terrestrial organisms. Further, their (eco)toxicity has been investigated, which for ILs depends on their specific structure, such as the head group or the side chain of the cation or the anion [8,9,10,11,12,13,14]. While significant biological activity might be unfavourable in cases where ILs are utilised as solvents or catalysts, their toxicity can be considered valuable for pharmaceutical and medical uses. In specific contexts, the antimicrobial attributes of ILs can be harnessed, and their efficacy against antibiotic-resistant pathogens has already been demonstrated [2,5,15,16,17,18,19]. The combinatorial modification of the cation–anion composition and additional modification of the chemical structure of each ion allow adjustments to the expected toxicity of ILs towards a particular pathogen. This specific feature of ILs could offer a significant benefit given the observed bacterial resistance to antibiotics [11,18,20,21].

Currently, most IL toxicity studies are based on determining toxicity values, such as minimal inhibitory concentrations (MICs) or half effective concentrations (EC_50_) for structurally similar ILs, with the final goal of developing a “quantitative structure–activity relationship” (QSAR) model [9,10,11,22,23,24,25]. Studies were performed at almost all trophic levels, and bacteria are still mainly used because of their extensive environmental, ecological and industrial relevance, short generation times and rapid growth [1,26]. Initially, it has been proposed that the primary mode of action of ILs, particularly those with elongated alkyl side chains, involves disrupting cell membrane function or even compromising cell integrity. However, it has been demonstrated that ILs can also engage with essential cellular elements such as proteins and DNA, inducing oxidative stress and disrupting metabolic functions [18]. Recent applications of ILs as “active” antimicrobials, disinfectants or pharmaceutical ingredients have also intensified the need for reliable “structure–activity relationships” (SARs) such as the well-known cationic alkyl side-chain effect, the number of alkyl side chains, and fluorinated anions or chaotropic anions [12,13,14,26,27,28].

One way to study how antimicrobials work is to perform molecular response analysis using Fourier-transform infrared (FTIR) spectroscopy. The approach entails identifying bacterial molecular patterns that mirror the general makeup of essential cellular biochemical components, observed as overlapping absorption bands. This method necessitates brief acquisition times and is cost-effective to operate, making it well suited as a high-throughput molecular fingerprinting technique [29,30,31]. Due to its substantial discriminatory capability, this approach has been effectively employed for identifying and differentiating species. It is a valuable tool for tracking phenotypic changes arising from environmental and biological disturbances [32,33,34]. Using FTIR molecular response spectroscopy, it has become possible to identify different modes of action [35,36,37,38] as well as ante-mortem responses of bacteria against antimicrobials, highlighting the adaptive diversity of bacterial stress responses [39]. Moreover, we found experimental evidence contrary to the previously predominant belief that the alkyl side-chain effect could be solely explained by an increasing disturbance of cell membrane function (and thus increasing toxicity) with increasing IL side-chain length [40,41].

In this study, a total of 18 different ILs covering three different antimicrobial active motifs were tested based on a previous study. The motifs of interest included ILs with a singular elongated alkyl side chain, the presence of multiple elongated side chains and fluorinated anions, as these had shown promising results in overcoming bacterial biocidal resistance [1,16,18,42]. In the case of ILs with elongated or multiple side chains, membrane disruption is the suggested mode of action, while fluorinated ILs are reported to also interact with and destabilise the cell wall [18]. For 18 different ILs, dose–response experiments and FTIR-based molecular response analysis for two human pathogens of high relevance, namely the Gram-negative enteropathogenic *E. coli* (EPEC) and Gram-positive methicillin-resistant *Staphylococcus aureus* (MRSA), were performed.

## 2. Results and Discussion

### 2.1. Determination of Ionic Liquid Minimal Bactericidal Concentrations

The present study evaluated the antimicrobial activity of 18 different ILs on two bacterial pathogens. The MBC (99.9% CFU reduction within one hour of exposure) values for each IL and bacterial strain are listed in Table 1. The individual mortality for each concentration of each IL can be found in Supplemental Tables S1–S6.

For cations with long alkyl side chains, the antimicrobial activity (MBC) increased with increasing side chain length, known as the cationic alkyl side chain effect [14]. Also, cations with two [DC_8_DMA][Cl] or three [TC_8_MA][Cl] side chains showed high antimicrobial activity against both bacterial species, which is in accordance with previously published results [8,16]. In the case of ILs with a relatively non-toxic cation but antimicrobial active anion, a set of fluorinated anions was chosen due to previous reports of antimicrobial activity [8,27]. All those ILs showed antimicrobial activity within the experimental setup of a one-hour exposure, with [BF_4_]^−^ the being most effective with an MBC of 125 mM for both bacterial species, EPEC and MRSA.

Overall, the results of the antimicrobial activity testing are in good accordance with published trends. For 14 of the 18 evaluated ILs, dose–response spectra reaching the MBC could be collected and further analysed, while for four ILs, the MBC was not reached at the highest tested concentration.

### 2.2. Molecular Response Analysis for EPEC

Molecular response values for all 18 tested ionic liquids for the protein and lipid spectral region at the lowest concentration, killing 99.9% of *E. coli* cells, are listed in Table 2. Despite the structural differences and the significant antimicrobial activity (ranging from 0.1 mM to >1000 mM) for each of the 18 ILs, a significantly higher molecular response was found in the protein region compared to the lipid region. While broad spectral regions were chosen for response analysis in previous studies performed by Corte et al. as well as our group [39,41], in this study, the most distinct concentration-dependent changes could be narrowed down to two spectral windows, namely 2935–2915 cm^−1^ for the lipid region and 1690–1620 cm^−1^ for the protein region (Table 1 and Table 2). All molecular response values for each IL, bacteria and additional spectral regions are listed in Supplemental Tables S1–S6.

**Figure 1:**
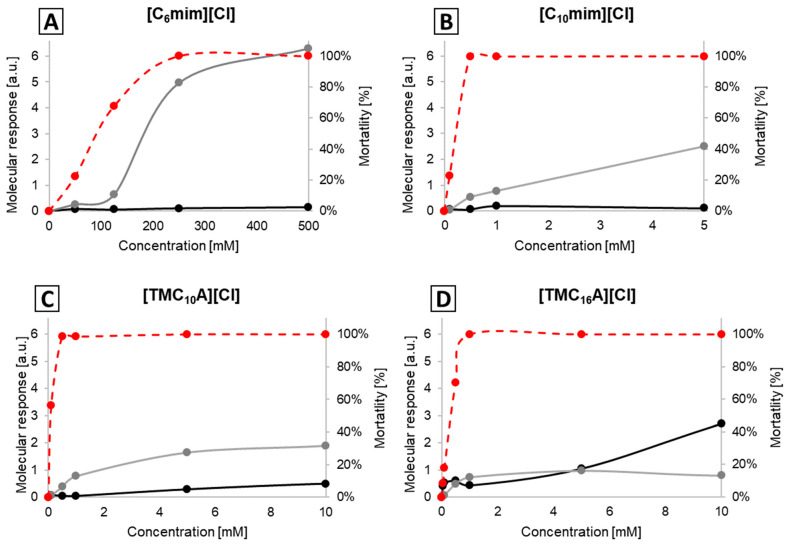
Molecular response indices of EPEC cells subjected to the action of [C_6_mim][Cl] (**A**), [C_10_mim][Cl] (**B**), [TMC_10_A][Cl] (**C**) and [TMC_16_A][Cl] (**D**). a.u. stands for “arbitrary units”; the grey line represents the spectral region of 1690–1620 cm^−1^ (Amide I), and the black line 2935–2915 cm^−1^ (fatty acids); the dashed red line represents mortality.

In the case of ILs with one elongated side chain ([C_n_mim]Cl and [TMC_n_A]Cl with n: 1–16), there is a clear correlation between the toxicity of the respective IL and the molecular response value in the protein region, while only a minimal molecular response can be observed in the lipid spectral region. Contrary to previous hypotheses, no correlation between cell mortality and changes in cellular membranes for ILs with alkyl side chains shorter than 16 was found, indicating that disruption of cell membranes is not the mode of action of these ILs. Even for [TMC_16_A][Cl] at the MBC, the dominant molecular responses are in the protein spectral region, while only at concentrations above the MBC is this trend switched, with a strong correlation between increasing toxicity and increasing molecular response value in the lipid region and only a weak response in the protein region. These findings are confirmed by the analysis of the present results, exemplarily shown in Figure 1 for EPEC and listed in detail in Tables S4–S6.

**Figure 2:**
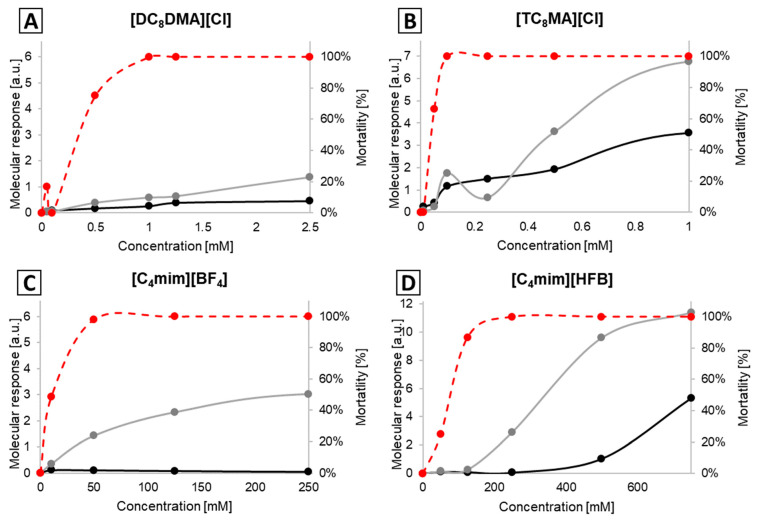
Molecular response indices of EPEC cells subjected to the action of [DC_8_DMA][Cl] (**A**), [TC_8_MA][Cl] (**B**), [C_4_mim][BF_4_] (**C**) and [C_4_mim][HFB] (**D**). a.u. stands for “arbitrary units”; the grey line represents the spectral region of 1690–1620 cm^−1^ (Amide I), and the black line 2935–2915 cm^−1^ (fatty acids); the dashed red line represents mortality.

In this study, we also systematically analysed ILs with either two or three elongated side chains as well as ILs with fluorinated anions having antimicrobial activity. Due to the overall similarity of results, exemplary results are depicted in Figure 2, while the detailed results are listed in Supplemental Tables S4–S6. Similar to the results obtained for ILs with one elongated alkyl side chain, most molecular responses were observed in the protein region, while the lipid region showed either a significantly smaller effect or no effect at all. These results indicate that such ILs primarily act on the protein region of the bacterial cell and not on the lipid region of the membrane. Overall, we could show that in the case of EPEC, only very few ILs showed any influence on the lipid spectral region, even at higher concentrations. Only in the case of [TC_8_MA][Cl] and [C_4_mim][HFB] could a significant molecular response be detected in the lipid region. However, it was still lower compared to the protein region and occurred only at concentrations above the respective MBC. It is thus unclear if this response could be considered a mode of action of the respective IL’s antimicrobial activity. To the best of our knowledge, only one other study so far has investigated the mechanism of action of ILs against *E. coli*. In this study, Ibsen et al. found a disruption of the lipid bilayer of choline-based ILs using flow cytometry [47]. However, the investigated substances were fundamentally different from the ILs included in this study, and thus, it is unclear if this contradicts or confirms the findings of this study. These results highlight again the need for further studies to improve the experimental knowledge regarding the mode of action of ILs. This information is crucial for assisting and improving current toxicity calculation approaches (QSAR), enhancing the development of ILs with active pharmaceutical ingredient motifs (API-IL) or utilising ILs as powerful antimicrobials.

### 2.3. Molecular Response Analysis for MRSA

Table 1 shows the molecular response values for all tested 18 ILs for the protein and lipid spectral region at the lowest concentration killing 99.9% *S. aureus* cells. Contrary to the results obtained for EPEC, molecular responses were found in both the protein and the lipid spectral regions for MRSA. These results indicate that the tested ionic liquids would act on cytoplasmic and cell-envelope-associated proteins and the lipids of the cellular membrane of *S. aureus*. However, a more detailed look at the dose–response spectra reveals a more complicated picture.

**Figure 3:**
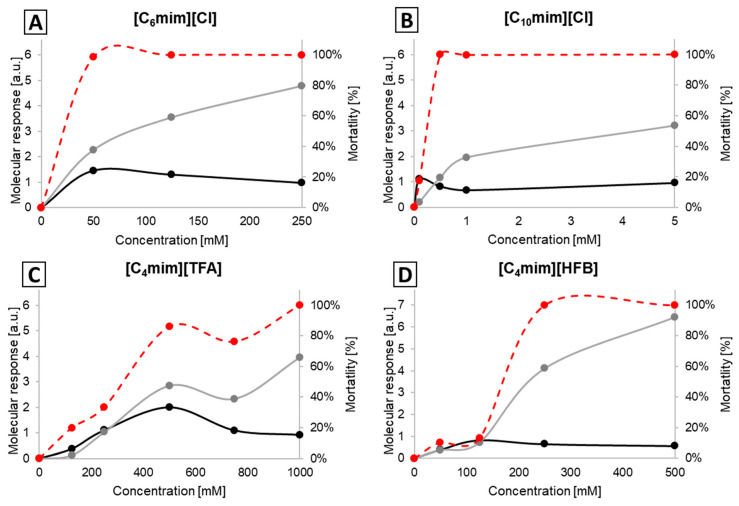
Molecular response indices of MRSA cells subjected to the action of [C_6_mim]][Cl] (**A**), [C_10_mim]][Cl] (**B**), [C_4_mim][TFA] (**C**) and [C_4_mim][HFB] (**D**). a.u. stands for “arbitrary units”; the grey line represents the spectral region of 1690–1620 cm^−1^ (Amide I), and the black line 2935–2915 cm^−1^ (fatty acids); the dashed red line represents mortality.

In the case of [C_n_mim][Cl]-based ILs as well as [C_4_mim][HFB] and [C_4_mim][TFA], the results are very similar to the ones for *E. coli*. For each of the five ILs, the molecular response at or above the MBC is dominated by changes to the protein region that correlate well with the observed toxicity, while the lipid spectral region is less affected (Figure 3; Tables S1–S3).

**Figure 4:**
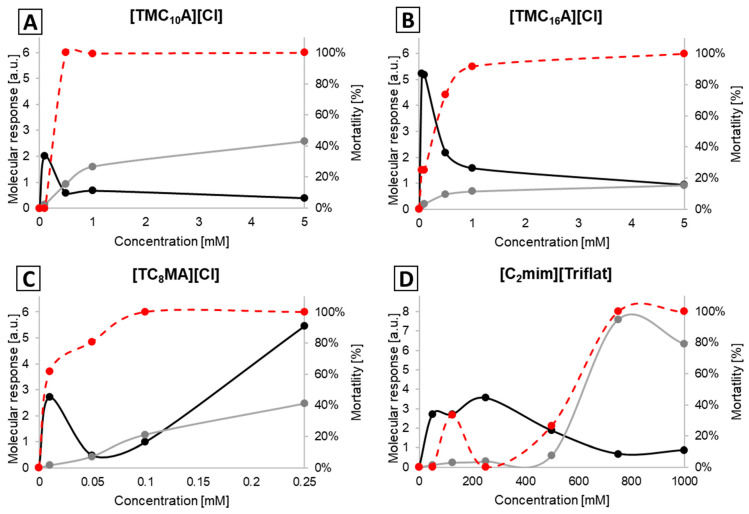
Molecular response indices of MRSA cells subjected to the action of [TMC_10_A][Cl] (**A**) [TMC_16_A][Cl] (**B**), [TC_8_MA][Cl] (**C**), [C_2_mim][Triflat] (**D**). a.u. stands for “arbitrary units”; the grey line represents the spectral region of 1690–1620 cm^−1^ (Amide I), and the black line 2935–2915 cm^−1^ (fatty acids); the dashed red line represents mortality.

In case of the [TMC_n_A][Cl]-based ILs as well as [C_4_mim][BF_4_], [C_4_mim][PF_6_] and [C_2_mim][Triflat], we also observe a clear correlation between toxicity and the molecular responses in the protein spectral region (see Supplementary Material Tables S2–S4), which also indicates a similar mode of action to that previously described [40,41]. However, in the case of several ILs, we could detect a significant ante-mortem response at sub-lethal concentrations of ILs. To our big surprise, our data revealed a significant molecular response in the spectral region containing fatty acids for MRSA exposed to sub-lethal concentrations of most of the ammonium-based ILs as well as [C_4_mim][BF_4_], [C_4_mim][PF_6_] and [C_2_mim][Triflat]. The response is not only more pronounced but also higher than the molecular response to even higher IL concentrations (Figure 4).

**Figure 5:**
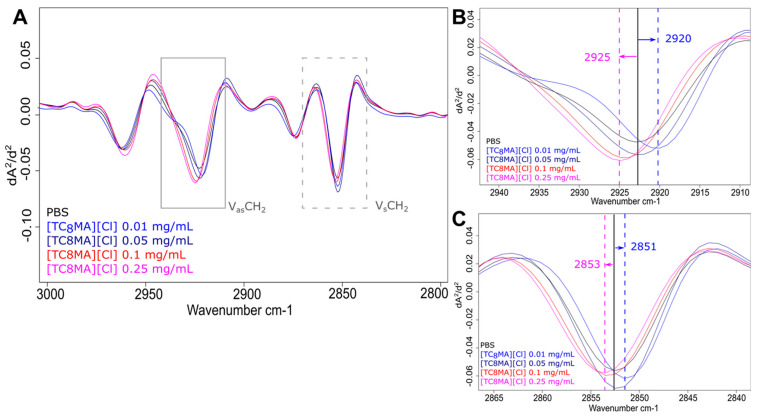
Effects of different IL treatments in the spectral range of fatty acids. FTIR spectra of MRSA treated with varying concentrations of [TC_8_MA][Cl] in the spectral range of 3000 to 2800 cm^−1^ (**A**). Short-term treatment with [TMC_8_MA][Cl] resulted in higher frequencies of peak maxima of ν_as_(CH_2_) (**B**) and ν_s_ (CH_2_) (**C**) compared to PBS control. The black line indicates peak maximum of the control and dashed lines indicated peak shifts.

**Figure 6:**
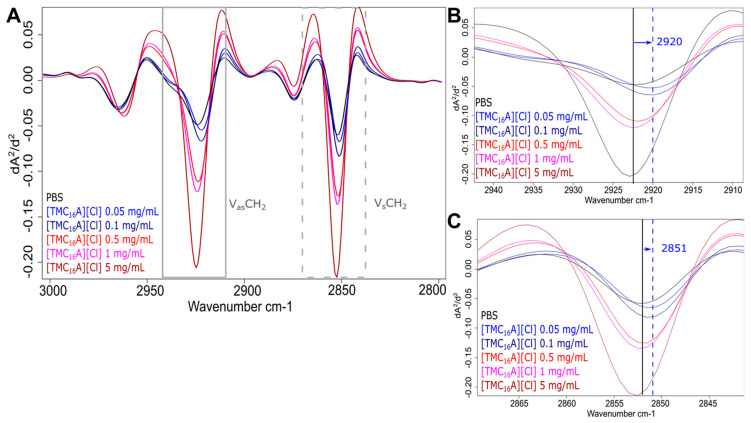
Effects of different IL treatments in the spectral range of fatty acids. FTIR spectra of MRSA treated with varying concentrations of [TMC_16_A][Cl] in the spectral range of 3000 to 2800 cm^−1^ (**A**). Short-term treatment with [TMC_16_A][Cl] resulted in higher frequencies of peak maxima of ν_as_(CH_2_) (**B**) and ν_s_ (CH_2_) (**C**) compared to PBS control. The black line indicates peak maximum of the control and dashed lines indicated peak shifts.

Spectral analysis revealed that the strong ante-mortem response is connected to a decreasing frequency in the asymmetric ν_as_(CH_2_) (near 2923 cm^−1^) and symmetric ν_s_(CH_2_) (near 2852 cm^−1^) stretching mode of CH_2_ functional groups in fatty acids. The effects on both stretching modes were identical, although the decrease in frequency was more pronounced for all four ILs in the asymmetric ν_as_(CH_2_) stretching mode (Figure 5 and Figure 6 and Table S7). Changes in the frequency of the conformation-sensitive ν_s_(CH_2_) and ν_as_(CH_2_) bands have been utilised to monitor the fluidity of biological membranes [48]. Studies using bacteria demonstrated that an elevation in the peak frequency of the ν_s_(CH_2_) and ν_as_(CH_2_) bands serves as an indicator of the fluidity of the membrane. Conversely, a decrease in the peak frequency suggests a decrease in membrane fluidity [49,50]. Thus, we assume that the reduction in peak frequency before bacterial death is related to a shift from disordered (“fluid”) to ordered (“rigid”) acyl chain conformations in phospholipid bilayers. However, the overall membrane disruption due to increasing IL concentrations leads to the opposite change in the lipid bilayer, showing an increase in membrane fluidity due to cell death. In *S. Typhimurium* and *S. enteritidis*, a drop in peak frequency and an associated decrease in membrane fluidity due to increased growth temperature and acidification were also detected [35,49]. This initial response to sub-lethal concentrations seems reasonable, as it would shield the bacteria from harmful environmental influences with a “protective coating”.

It is known that Gram-positive bacteria, such as MRSA, modify their membrane composition to cope with challenging environmental conditions, such as quaternary ammonium compounds [39], which are structurally similar to some of the antimicrobial active ILs. Thus, it is tempting to speculate that MRSA modify their cell membrane structure to defend against the stress exerted by the ILs.

The presented results also confirm previous differences between Gram-positive and Gram-negative bacteria, which have been previously reported solely based on toxicity data [8,51]. As such changes at sub-lethal concentrations are not observed for EPEC, it is likely that they can activate different stress response mechanisms such as LPS modifications, efflux pumps or SOS responses. In contrast, NRSA can quickly change their membrane to improve its permeability and increase their tolerance against ILs [52]. However, the following limitations should be considered for the interpretation of the study and addressed in future research. The investigations of membrane fluidity using FTIR spectroscopy are well documented; however, since many factors can influence membrane substructures, further investigations are necessary to identify the mechanisms of bacterial adaptations to ILs. This could include the analysis of knock-out mutants of known bacterial efflux pumps and SOS regulators, as well as using other methods for investigating membrane fluidity and permeabilisation (e.g., microscopic visualisation of fluorescent membrane dyes) [53]. Moreover, the study results refer to only one species/strain per Gram type, and although they are frequently studied pathogens, they are linked to species- or subtype-specific adaptations to ILs. Thus, the hypothesis that Gram-positive bacteria rapidly adapt their membrane to resist ILs must be investigated further to see if this generally applies to all Gram-positive bacteria by testing additional relevant species. It would also be very interesting to examine the speed of the observed adaptations as well as if bacteria would demonstrate a co-tolerance against other antimicrobials. Currently, it is also unclear why the effect was not observed for all ILs, and a more diverse set should be investigated to examine this point. Remarkably, such changes could be actually observed directly by FTIR spectroscopy, which once again demonstrates the advantage of this method in following the overall biochemical composition on a cellular level. Finally, a better understanding of the mode of action of ILs could benefit public health in combating various pathogens by providing alternative strategies against bacterial resistance. ILs could potentially target resistant pathogens in medical and industrial environments, e.g., by being used in surface coatings to develop anti-biofilm materials that combat bacterial growth on medical devices or in food production. In addition, ILs can act as counterions for pharmaceuticals as API-ILs introducing a secondary mode of action to create synergistic and complementary effects against bacteria. Therefore, it is crucial to understand cellular changes induced by specific IL motifs such as changed membrane composition or fluidity as they could alter the effectivity of antibiotics. Future research should also expand to investigating the environmental fate and biodegradability of any potential antimicrobial IL used as a biocide or as a part of API-ILs.

## 3. Materials and Methods

### 3.1. Ionic Liquids

**Figure 7:**
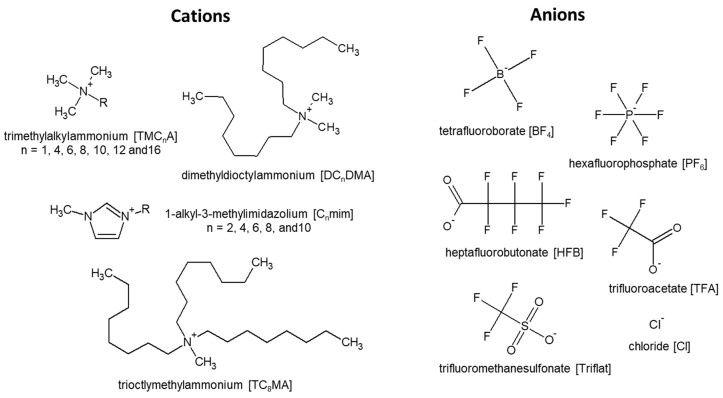
Graphic representation of cations and anions comprising the 18 ionic liquids investigated in this study.

A total of 18 different ILs covering three different antimicrobial active motifs were tested (Figure 7). The ILs containing one elongated cationic alkyl side chain were based on either 1-alkyl-3-methylimidazolium chloride ([C_n_mim][Cl] (n = 2, 4, 6, 8 and 10)) or trimethylalkylammonium chloride ([TMC_n_A][Cl] (n = 1, 4, 8, 10, 12 and 16)). In addition, two ILs with multiple elongated alkyl side chains were investigated, namely dioctyldimethylammonium chloride ([DC_8_DMA][Cl]) and trioctylmethylammonium chloride ([TC_8_MA][Cl]). As representatives of antimicrobial active ILs with fluorinated anions, a set of five ILs with non-toxic cations were included; 1-butyl-3-methylimidazolium tetrafluoroborate ([C_4_mim][BF_4_]), 1-butyl-3-methylimidazolium heptafluorobutonate ([C_4_mim][HFB]), 1-butyl-3-methylimidazolium hexafluorophosphate ([C_4_mim][PF_6_]), 1-butyl-3-methylimidazolium trifluoroacetate ([C_4_mim][TFA]) and 1-ethyl-3-methylimidazolium trifluoromethanesulfonate ([C_2_mim][Triflat]). Fourteen of the eighteen ILs were provided by Proionic GmbH (Grambach, Austria) with a nominal purity higher than 95%, while [TMCnA][Cl] (n = 8, 10, 12 and 16) were synthesised using the CBILS^®^ route [54,55]. The precursor ILs used for synthesis ([TMC_n_A][MC] with n = 8, 10, 12 and 16) were provided by Proionic GmbH (Grambach, Austria). Hydrochloric acid was obtained from Merck KGaA (Darmstadt, Germany).

### 3.2. Bacterial Strains and Growth Conditions

The *eae*-positive enteropathogenic *E. coli* O8:H14 strain (EPEC) obtained from AGES (Vienna, Austria) and community-associated methicillin-resistant *S. aureus* (CA-MRSA) strain NRS384 (also known as USA300-0114) obtained from the Network on Antimicrobial Resistance in *Staphylococcus aureus* (NARSA) collection were maintained at −80 °C using MicroBank^TM^ technology (Pro-Lab Diagnostics, Richmont Hill, ON, Canada). All bacterial strains were grown overnight in tryptone soya broth with 0.6% (*w*/*v*) yeast extract (TSB-Y; Oxoid, Basingstoke, Hampshire, UK) at 37 °C.

### 3.3. FTIR Spectroscopy Procedure

For FTIR measurements, bacterial cells were centrifuged (5 min at 8000× *g*), washed two times with PBS buffer and resuspended in 1 mL of the respective test solution with a final optical density of OD_610_ = 5. Non-treatment control cells were resuspended directly in PBS. All tests were performed in triplicate in three independent experiments on different days, including non-treatment controls. The resuspended cells were incubated for one hour at 30 °C in a shaking incubator set at 250 rpm. Afterward, each sample was centrifuged (5 min at 8000× *g*), washed twice with 1 mL PBS to remove the remaining ionic liquid and resuspended in 100 µL PBS to obtain a final OD_610_ = 50 [39,40]. From the same sample, three independent FTIR measurements with 30 μL each and biocidal activity determination were performed. FTIR spectroscopy was conducted utilising a TENSOR 27 FTIR spectrometer equipped with an HTS-XT accessory for rapid automation of the analysis (BRUKER Optics GmbH, Ettlingen, Germany), and measurements were performed in transmission mode. All spectra were recorded in the range between 4000 and 500 cm^−1^ using the following parameters: 6 cm^−1^ spectral resolution, zero-filling factor 4, Blackmann–Harris 3-term apodisation and 32 interferograms averaged with background subtraction (blank control) for each spectrum [33].

### 3.4. Spectral Pre-Processing and Molecular Response Analysis

OPUS software version 7.2 (BRUKER Optics GmbH, Ettlingen, Germany) was used for spectral evaluation and pre-processing, including the calculation of second derivatives of the original spectra with a 9-point Savitzky–Golay filter and subsequent vector normalisation [33,56]. The spectral regions that offered the information for the following classes of cellular macromolecules were examined: fatty acids (3000 to 2800 cm^−1^) and proteins (1800 to 1500 cm^−1^). For the analysis of the 18 different ILs on two bacterial pathogens, molecular response values for selected spectral regions were determined by calculating the spectral distances (d-value) using OPUS software version 7.2 [57]. The d-value represents the area of difference between two spectral curves being compared [43]. The greater the variance between two spectra within the tested frequency ranges, the higher the spectral distance. In this approach, each FTIR spectrum of bacterial cells exposed to an IL at a given concentration was compared against the respective non-treatment control of the respective experiment, and the molecular differences were quantitatively described by the d-value. Technical triplicates were averaged per experiment, and the respective means of at least two experiments for each IL concentration were calculated to analyse concentration-dependent relationships. We calculated the respective d-values for four distinct spectral regions (Table 2), which have been demonstrated to be primarily impacted by ILs in our previous studies [30,40,41,44,45,46,58].

### 3.5. Biocidal Activity Test

The biocidal activity tests were performed in parallel with the FTIR spectroscopy-based bioassay to compare metabolomic damages with loss of viability. From each cell suspension prepared for FTIR analysis, 10 μL was serially diluted onto TSA+Y plates to determine viable cell counts. The biocidal effect of the tested ILs at different concentrations was calculated as the mortality rate (%) compared to the CFU number in the phosphate-buffered saline (PBS; NaCl [137 mM], KCl [2.7 mM], Na_2_HPO_4_ [10 mM] and KH_2_PO_4_ [2 mM] adjusted to pH 7.4) control sample.

## 4. Conclusions

In this study, we evaluated the potential of FTIR spectroscopy as a high-throughput method for monitoring the response of bacterial cells treated with ILs. Dose–response investigations of 18 structurally different ILs revealed insights into the mode of action of their antimicrobial activity as well as ante-mortem stress responses of two important bacterial pathogens. Our results indicate that the effect of ILs on biological membranes can only be attributed to alkyl side chains > 12, whereas ILs with shorter side chains, multiple side chains and highly fluorinated anions interact with the proteins of the bacterial cells. Furthermore, our study provides evidence for an ante-mortem response of MRSA against certain ILs, indicated by the adaptive response before death. Contrary to the molecular response at cell death (MBC), the ante-mortem response is solely observed in the lipid region of the bacterial cell. This indicates a change in the overall lipid composition of the membrane in order to reduce the permeability towards the ILs. Interestingly, as the effect was not observable for all investigated active ILs, structurally optimised antimicrobial ILs that do not trigger an ante-mortem response can be envisioned.

## Figures and Tables

**Table 1:** Minimal bactericidal concentration and molecular response indices of 18 ILs for EPEC and MRSA.

	*Staphylococcus aureus* (MRSA)			*Escherichia coli* (EPEC)		
	MBC [mg/mL]	d-Values for 2935–2915 cm^−1^	d-Values for 1690–1620 cm^−1^	MBC [mg/mL]	d-Values for 2935–2915 cm^−1^	d-Values for 1690–1620 cm^−1^
[C_2_mim][Cl]	>1000	0.21	0.32	>1000	0.05	0.11
[C_4_mim][Cl]	>1000	1.26	2.93	>1000	0.08	2.14
[C_6_mim][Cl]	125	1.30	3.54	250	0.11	4.97
[C_8_mim][Cl]	5	1.00	2.24	50	0.15	2.94
[C_10_mim][Cl]	0.5	0.82	1.16	5	0.11	2.51
[TMA][Cl]	>1000	2.25	0.15	1000	1.83	3.32
[TMC_4_A][Cl]	>1000	4.35	0.39	750	0.06	3.87
[TMC_8_A][Cl]	25	1.08	1.74	50	0.09	1.55
[TMC_10_A][Cl]	5	0.40	2.57	5	0.29	1.65
[TMC_12_A][Cl]	5	0.55	2.68	5	0.06	1.25
[TMC_16_A][Cl]	5	0.94	0.92	1	0.44	0.73
[TC_8_MA][Cl]	0.1	0.98	1.27	0.1	1.17	1.76
[DC_8_DMA][Cl]	1	0.97	1.23	1.25	0.39	0.65
[C_4_mim][BF_4_]	125	1.76	2.54	125	0.08	2.32
[C_4_mim][HFB]	250	0.65	4.11	250	0.05	2.92
[C_4_mim][PF_6_]	750	0.74	2.98	500	0.14	1.15
[C_4_mim][TFA]	1000	0.92	3.95	500	0.13	2.32
[C_2_mim][Triflat]	750	0.69	7.58	750	0.19	9.02

**Table 2:** Assignment of bands of FTIR spectra mentioned in this publication.

Frequency (cm^−1^)	Assignment	References
3000–2800	Fatty acid region; dominated by stretching vibrations of functional groups usually present in the fatty acid components of the bacterial membrane	[43]
~2923	C-H str (*asym*) of CH_2_ functional groups in fatty acids	[30,44]
~2852	C-H str (*sym*) of CH_2_ functional groups in fatty acids	[30,44]
1800–1500	Amide region; dominated by the amide bands of proteins and peptides	[43]
~1638	Amide I of β-pleated sheet	[30]
~1630–1620	Changes in β-pleated sheet conformation caused by misfolding and/or protein aggregation	[45,46]

*sym*, symmetrical; *asym*, asymmetrical.

## Data Availability

Data are contained within the article and Supplementary Materials.

## Supplementary Materials

The following supporting information can be downloaded at https://www.mdpi.com/article/10.3390/ijms25094705/s1.

## References

[1] Egorova K.S., Gordeev E.G., Ananikov V.P. (2017). Biological Activity of Ionic Liquids and Their Application in Pharmaceutics and Medicine. Chem. Rev..

[2] Ferraz R., Branco L.C., Prudencio C., Noronha J.P., Petrovski Z. (2011). Ionic Liquids as Active Pharmaceutical Ingredients. ChemMedChem.

[3] Hough W.L., Smiglak M., Rodriguez H., Swatloski R.P., Spear S.K., Daly D.T., Pernak J., Grisel J.E., Carliss R.D., Soutullo M.D. (2007). The Third Evolution of Ionic Liquids: Active Pharmaceutical Ingredients. New J. Chem..

[4] Md Moshikur R., Chowdhury M.R., Moniruzzaman M., Goto M. (2020). Biocompatible Ionic Liquids and Their Applications in Pharmaceutics. Green Chem..

[5] Ferraz R., Silva D., Dias A.R., Dias V., Santos M.M., Pinheiro L., Prudêncio C., Noronha J.P., Petrovski Ž., Branco L.C. (2020). Synthesis and Antibacterial Activity of Ionic Liquids and Organic Salts Based on Penicillin g and Amoxicillin Hydrolysate Derivatives against Resistant Bacteria. Pharmaceutics.

[6] Wu X., Zhu Q., Chen Z., Wu W., Lu Y., Qi J. (2021). Ionic Liquids as a Useful Tool for Tailoring Active Pharmaceutical Ingredients. J. Control. Release.

[7] Pedro S.N., Freire C.S.R., Silvestre A.J.D., Freire M.G. (2020). The Role of Ionic Liquids in the Pharmaceutical Field: An Overview of Relevant Applications. Int. J. Mol. Sci..

[8] Weyhing-Zerrer N., Gundolf T., Kalb R., Oßmer R., Rossmanith P., Mester P. (2017). Predictability of Ionic Liquid Toxicity from a SAR Study on Different Systematic Levels of Pathogenic Bacteria. Ecotoxicol. Environ. Saf..

[9] Ghanem O.B., Mutalib M.I.A., Leveque J.M., El-Harbawi M. (2017). Development of QSAR Model to Predict the Ecotoxicity of Vibrio Fischeri Using COSMO-RS Descriptors. Chemosphere.

[10] Ghanem O.B., Papaiconomou N., Bdul Mutalib M.I., Viboud S., El-Harbawi M., Uemura Y., Gonfa G., Azmi Bustam M., Leveque J.M. (2015). Thermophysical Properties and Acute Toxicity towards Green Algae and Vibrio Fischeri of Amino Acid-Based Ionic Liquids. J. Mol. Liq..

[11] Wyrzykowska E., Rybińska-Fryca A., Sosnowska A., Puzyn T. (2019). Virtual Screening in the Design of Ionic Liquids as Environmentally Safe Bactericides. Green Chem..

[12] Mester P., Wagner M., Rossmanith P. (2015). Antimicrobial Effects of Short Chained Imidazolium-Based Ionic Liquids-Influence of Anion Chaotropicity. Ecotoxicol. Environ. Saf..

[13] Matzke M., Stolte S., Thiele K., Juffernholz T., Arning J., Ranke J., Welz-Biermann U., Jastorff B. (2007). The Influence of Anion Species on the Toxicity of 1-Alkyl-3- Methylimidazolium Ionic Liquids Observed in an (Eco)Toxicological Test Battery. Green Chem..

[14] Stolte S., Matzke M., Arning J., Boschen A., Pitner W.R., Welz-Biermann U., Jastorff B., Ranke J. (2007). Effects of Different Head Groups and Functionalised Side Chains on the Aquatic Toxicity of Ionic Liquids. Green Chem..

[15] Kemp T.J. (2012). Ionic Liquids—Pharmaceutical Potential. Sci. Prog..

[16] Gundolf T., Rauch B., Kalb R., Rossmanith P., Mester P. (2018). Influence of Bacterial Lipopolysaccharide Modifications on the Efficacy of Antimicrobial Ionic Liquids. J. Mol. Liq..

[17] Guo J., Qian Y., Sun B., Sun Z., Chen Z., Mao H., Wang B., Yan F. (2019). Antibacterial Amino Acid-Based Poly(Ionic Liquid) Membranes: Effects of Chirality, Chemical Bonding Type, and Application for MRSA Skin Infections. ACS Appl. Bio Mater..

[18] Michalski J., Odrzygóźdź C., Mester P., Narożna D., Cłapa T. (2023). Defeat Undefeatable: Ionic Liquids as Novel Antimicrobial Agents. J. Mol. Liq..

[19] Cłapa T., Michalski J., Syguda A., Narożna D., van Oostrum P., Reimhult E. (2021). Morpholinium-Based Ionic Liquids Show Antimicrobial Activity against Clinical Isolates of Pseudomonas Aeruginosa. Res. Microbiol..

[20] Ferraz R., Branco L.C., Marrucho I.M., Araújo J.M.M., Rebelo L.P.N., Da Ponte M.N., Prudêncio C., Noronha J.P., Petrovski E. (2012). Development of Novel Ionic Liquids Based on Ampicillin. MedChemComm.

[21] Araújo J.M.M., Florindo C., Pereiro A.B., Vieira N.S.M., Matias A.A., Duarte C.M.M., Rebelo L.P.N., Marrucho I.M. (2014). Cholinium-Based Ionic Liquids with Pharmaceutically Active Anions. RSC Adv..

[22] Cvjetko Bubalo M., Radosevic K., Radojcic Redovnikovic I., Halambek J., Gaurina Srcek V. (2014). A Brief Overview of the Potential Environmental Hazards of Ionic Liquids. Ecotoxicol. Environ. Saf..

[23] Heckenbach M.E., Romero F.N., Green M.D., Halden R.U. (2016). Meta-Analysis of Ionic Liquid Literature and Toxicology. Chemosphere.

[24] Lotfi S., Ahmadi S., Zohrabi P. (2020). QSAR Modeling of Toxicities of Ionic Liquids toward *Staphylococcus aureus* Using SMILES and Graph Invariants. Struct. Chem..

[25] Grzonkowska M., Sosnowska A., Barycki M., Rybinska A., Puzyn T. (2016). How the Structure of Ionic Liquid Affects Its Toxicity to Vibrio Fischeri?. Chemosphere.

[26] Ranke J., Molter K., Stock F., Bottin-Weber U., Poczobutt J., Hoffmann J., Ondruschka B., Filser J., Jastorff B. (2004). Biological Effects of Imidazolium Ionic Liquids with Varying Chain Lengths in Acute Vibrio Fischeri and WST-1 Cell Viability Assays. Ecotoxicol. Environ. Saf..

[27] Weyhing-Zerrer N., Kalb R., Oßmer R., Rossmanith P., Mester P. (2018). Evidence of a Reverse Side-Chain Effect of Tris(Pentafluoroethyl)Trifluorophosphate [FAP]-Based Ionic Liquids against Pathogenic Bacteria. Ecotoxicol. Environ. Saf..

[28] Forero-Doria O., Araya-Maturana R., Barrientos-Retamal A., Morales-Quintana L., Guzmán L. (2019). N-Alkylimidazolium Salts Functionalized with p-Coumaric and Cinnamic Acid: A Study of Their Antimicrobial and Antibiofilm Effects. Molecules.

[29] Naumann D., Helm D., Labischinski H. (1991). Microbiological Characterizations by FT-IR Spectroscopy. Nature.

[30] Naumann D. (2000). Encyclopedia of Analytical Chemistry.

[31] Vernocchi P., Vannini L., Gottardi D., Del Chierico F., Serrazanetti D.I., Ndagijimana M., Guerzoni M.E. (2012). Integration of Datasets from Different Analytical Techniques to Assess the Impact of Nutrition on Human Metabolome. Front. Cell. Infect. Microbiol..

[32] Ami D., Natalello A., Gatti-Lafranconi P., Lotti M., Doglia S.M. (2005). Kinetics of Inclusion Body Formation Studied in Intact Cells by FT-IR Spectroscopy. FEBS Lett..

[33] Grunert T., Monahan A., Lassnig C., Vogl C., Müller M., Ehling-Schulz M. (2014). Deciphering Host Genotype-Specific Impacts on the Metabolic Fingerprint of Listeria Monocytogenes by FTIR Spectroscopy. PLoS ONE.

[34] Wenning M., Scherer S. (2013). Identification of Microorganisms by FTIR Spectroscopy: Perspectives and Limitations of the Method. Appl. Microbiol. Biotechnol..

[35] Alvarez-Ordez A., Halisch J., Prieto M. (2010). Changes in Fourier Transform Infrared Spectra of Salmonella Enterica Serovars Typhimurium and Enteritidis after Adaptation to Stressful Growth Conditions. Int. J. Food Microbiol..

[36] Preisner O., Lopes J.A., Guiomar R., MacHado J., Menezes J.C. (2007). Fourier Transform Infrared (FT-IR) Spectroscopy in Bacteriology: Towards a Reference Method for Bacteria Discrimination. Anal. Bioanal. Chem..

[37] Wehrli P.M., Lindberg E., Svensson O., Sparen A., Josefson M., Dunstan R.H., Wold A.E., Gottfries J. (2014). Exploring Bacterial Phenotypic Diversity Using Factorial Design and FTIR Multivariate Fingerprinting. J. Chemom..

[38] Winder C.L., Goodacre R. (2004). Comparison of Diffuse-Reflectance Absorbance and Attenuated Total Reflectance FT-IR for the Discrimination of Bacteria. Analyst.

[39] Corte L., Tiecco M., Roscini L., De Vincenzi S., Colabella C., Germani R., Tascini C., Cardinali G. (2015). FTIR Metabolomic Fingerprint Reveals Different Modes of Action Exerted by Structural Variants of N-Alkyltropinium Bromide Surfactants on *Escherichia coli* and Listeria Innocua Cells. PLoS ONE.

[40] Mester P., Jehle A.K., Leeb C., Kalb R., Grunert T., Rossmanith P. (2016). FTIR Metabolomic Fingerprint Reveals Different Modes of Action Exerted by Active Pharmaceutical Ingredient Based Ionic Liquids (API-ILs) on: Salmonella Typhimurium. RSC Adv..

[41] Mester P., Robben C., Witte A.K., Kalb R., Ehling-Schulz M., Rossmanith P., Grunert T. (2019). FTIR Spectroscopy Suggests a Revised Mode of Action for the Cationic Side-Chain Effect of Ionic Liquids. ACS Comb. Sci..

[42] Bromberger B., Sommer J., Robben C., Trautner C., Kalb R., Rossmanith P., Mester P.J. (2020). Evaluation of the Antimicrobial Activity of Pyrithione-Based Ionic Liquids. Sep. Purif. Technol..

[43] Naumann D., Labischinski H., Giesbrecht P., Nelson W.H. (1991). The Characterization of Microorganisms by Fourier-Transform Infrared Spectroscopy (FT-IR). Modem Techniques for Rapid Microbiological Analysis.

[44] Helm D., Labischinski H., Schallehn G., Naumann D. (1991). Classification and Identifictaion of Bacteria by Fourier-Transform Infrared Spectroscopy. J. Gen. Microbiol..

[45] Socrates G. (2004). Infrared and Raman Characteristic Group Frequencies: Tables and Charts.

[46] Miller L.M., Bourassa M.W., Smith R.J. (2013). FTIR Spectroscopic Imaging of Protein Aggregation in Living Cells. Biochim. Biophys. Acta.

[47] Ibsen K.N., Ma H., Banerjee A., Tanner E.E.L., Nangia S., Mitragotri S. (2018). Mechanism of Antibacterial Activity of Choline-Based Ionic Liquids (CAGE). ACS Biomater. Sci. Eng..

[48] Kóta Z., Debreczeny M., Szalontai B. (1999). Separable Contributions of Ordered and Disordered Lipid Fatty Acyl Chain Segments to VCH2 Bands in Model and Biological Membranes: A Fourier Transform Infrared Spectroscopic Study. Biospectroscopy.

[49] Scherber C.M., Schottel J.L., Aksan A. (2009). Membrane Phase Behavior of *Escherichia coli* during Desiccation, Rehydration, and Growth Recovery. Biochim. Biophys. Acta (BBA) Biomembr..

[50] Beney L., Mille Y., Gervais P. (2004). Death of *Escherichia coli* during Rapid and Severe Dehydration Is Related to Lipid Phase Transition. Appl. Microbiol. Biotechnol..

[51] Gundolf T., Weyhing-Zerrer N., Sommer J., Kalb R., Schoder D., Rossmanith P., Mester P. (2019). Biological Impact of Ionic Liquids Based on Sustainable Fatty Acid Anions Examined with a Tripartite Test System. ACS Sustain. Chem. Eng..

[52] Wang X., Chen Z., Mu Q., Wu X., Zhang J., Mao D., Luo Y., Alvarez P.J.J. (2020). Ionic Liquid Enriches the Antibiotic Resistome, Especially Efflux Pump Genes, before Significantly Affecting Microbial Community Structure. Environ. Sci. Technol..

[53] Scheinpflug K., Krylova O., Strahl H. (2017). Measurement of Cell Membrane Fluidity by Laurdan GP: Fluorescence Spectroscopy and Microscopy. Methods Mol. Biol..

[54] Kalb R.S., Stepurko E.N., Emel’yanenko V.N., Verevkin S.P. (2016). Carbonate Based Ionic Liquid Synthesis (CBILS[Registered Sign]): Thermodynamic Analysis. Phys. Chem. Chem. Phys..

[55] Kalb R., Wesner W., Hermann R., Kotschan M., Schelch M., Staber W. (2005). Verfahren zur Herstellung Ionischer Flüssigkeiten, Ionischer Feststoffe Oder Gemische Derselben.

[56] Grunert T., Wenning M., Barbagelata M.S., Fricker M., Sordelli D.O., Buzzola F.R., Ehling-Schulz M. (2013). Rapid and Reliable Identification of *Staphylococcus aureus* Capsular Serotypes by Means of Artificial Neural Network-Assisted Fourier Transform Infrared Spectroscopy. J. Clin. Microbiol..

[57] (2006). Bruker Manual Opus Spectroscopy Software, Version 6.

[58] Tidy R.J., Lam V., Fimognari N., Mamo J.C., Hackett M.J. (2017). FTIR Studies of the Similarities between Pathology Induced Protein Aggregation in Vivo and Chemically Induced Protein Aggregation Ex Vivo. Vib. Spectrosc..

