# Supplementary material for: Antimicrobial Ionic Liquids: Ante-Mortem Mechanisms of Pathogenic EPEC and MRSA Examined by FTIR Spectroscopy
Source: Int J Mol Sci. 2024 Apr 26;25(9):4705. doi: 10.3390/ijms25094705 (PMC11083031; doi:10.3390/ijms25094705)
Supplement: Supplementary file 1 [file ijms-25-04705-s001.zip › ijms-2942559-supplementary.pdf]

Table S1: Dose-Dependent Mortality Rates of MRSA exposed to [$C_n$mim][Cl] based ILs and corresponding molecular response values for four selected spectral regions.

IL name	IL concentration [mM]	Mortality [%]		d-values for 2860-2845 cm^{-1}		d-values for 2935-2915 cm^{-1}		d-values for 1570-1515 cm^{-1}		d-values for 1690-1620 cm^{-1}	
		Mean	SD	Mean	SD	Mean	SD	Mean	SD	Mean	SD
**[C_2mim] [Cl]**	0	**0.0%**	0.0%	**0.000**	0.000	**0.000**	0.000	**0.000**	0.000	**0.000**	0.000
	750	**56.3%**	25.5%	**0.195**	0.139	**0.534**	0.371	**0.268**	0.026	**0.330**	0.155
	1000	**58.6%**	17.2%	**0.084**	0.067	**0.213**	0.173	**0.364**	0.256	**0.317**	0.240
**[C_4mim] [Cl]**	0	**0.0%**	0.0%	**0.000**	0.000	**0.000**	0.034	**0.000**	0.000	**0.000**	0.000
	125	**9.5%**	0.0%	**0.004**	0.003	**0.047**	0.122	**0.062**	0.050	**0.034**	0.019
	250	**28.5%**	0.0%	**0.008**	0.009	**0.150**	0.000	**0.120**	0.099	**0.055**	0.034
	500	**20.6%**	12.4%	**0.052**	0.016	**0.742**	0.240	**0.302**	0.059	**0.308**	0.206
	750	**36.3%**	45.2%	**0.321**	0.322	**1.081**	0.935	**0.386**	0.230	**2.112**	2.123
	1000	**66.7%**	33.3%	**0.392**	0.316	**1.257**	0.835	**0.424**	0.263	**2.928**	2.420
**[C_6mim] [Cl]**	0	**0.0%**	0.0%	**0.000**	0.000	**0.000**	0.000	**0.000**	0.000	**0.000**	0.000
	50	**98.4%**	1.9%	**0.524**	0.190	**1.458**	0.825	**0.285**	0.092	**2.267**	0.247
	125	**100.0%**	0.1%	**0.475**	0.330	**1.301**	1.063	**0.559**	0.231	**3.539**	0.886
	250	**100.0%**	0.0%	**0.362**	0.368	**0.980**	1.097	**0.717**	0.108	**4.773**	1.222
**[C_8mim] [Cl]**	0	**0.0%**	0.0%	**0.000**	0.000	**0.000**	0.000	**0.000**	0.000	**0.000**	0.000
	1	**27.5%**	26.3%	**0.484**	0.271	**1.529**	0.983	**0.241**	0.028	**0.371**	0.122
	5	**99.9%**	0.1%	**0.341**	0.238	**0.996**	0.743	**0.300**	0.057	**2.238**	0.293
	12.5	**100.0%**	0.1%	**0.427**	0.181	**1.364**	0.746	**0.718**	0.244	**3.110**	1.059
**[C_{10}mim] [Cl]**	0	**0.0%**	0.0%	**0.000**	0.000	**0.000**	0.000	**0.000**	0.000	**0.000**	0.000
	0.1	**17.5%**	6.3%	**0.332**	0.330	**1.107**	1.187	**0.272**	0.076	**0.214**	0.063
	0.5	**99.9%**	0.1%	**0.302**	0.197	**0.822**	0.615	**0.209**	0.045	**1.165**	0.381
	1	**99.7%**	0.4%	**0.261**	0.152	**0.671**	0.409	**0.262**	0.074	**1.960**	0.357
	5	**100.0%**	0.0%	**0.402**	0.239	**0.958**	0.505	**0.627**	0.234	**3.214**	0.798

Table S2: Dose-Dependent Mortality Rates of MRSA exposed to [C_4mim][X] based ILs and corresponding molecular response values for four selected spectral regions.

IL name	IL concentration [mM]	Mortality [%] Mean	Mortality [%] SD	d-values for 2860-2845 cm^{-1} Mean	SD	d-values for 2935-2915 cm^{-1} Mean	SD	d-values for 1570-1515 cm^{-1} Mean	SD	d-values for 1690-1620 cm^{-1} Mean	SD
**[C_4mim] [BF_4]**	0	**0.0%**	0.0%	**0.000**	0.000	**0.000**	0.000	**0.000**	0.000	**0.000**	0.000
	10	**0.0%**	0.0%	**0.923**	0.587	**2.422**	1.809	**0.210**	0.059	**0.629**	0.323
	50	**72.2%**	41.7%	**0.972**	0.523	**2.424**	1.670	**0.337**	0.128	**1.558**	0.377
	125	**100.0%**	0.0%	**0.781**	0.396	**1.764**	1.085	**0.456**	0.093	**2.536**	0.500
**[C_4mim] [HFB]**	0	**0.0%**	0.0%	**0.000**	0.000	**0.000**	0.000	**0.000**	0.000	**0.000**	0.000
	50	**10.4%**	14.7%	**0.282**	0.176	**0.390**	0.115	**0.381**	0.111	**0.373**	0.015
	125	**13.0%**	18.3%	**0.399**	0.310	**0.829**	0.309	**0.380**	0.097	**0.722**	0.144
	250	**100.0%**	0.0%	**0.365**	0.368	**0.650**	0.644	**0.468**	0.365	**4.110**	0.847
	500	**100.0%**	0.0%	**0.233**	0.116	**0.562**	0.244	**1.036**	0.099	**6.461**	0.403
**[C_4mim] [PF_6]**	0	**0.0%**	0.0%	**0.000**	0.000	**0.000**	0.000	**0.000**	0.000	**0.000**	0.000
	50	**15.4%**	0.0%	**0.738**	0.688	**2.042**	1.679	**0.529**	0.427	**0.380**	0.279
	125	**44.9%**	21.8%	**0.551**	0.442	**1.689**	1.520	**0.322**	0.044	**0.547**	0.070
	250	**84.1%**	16.1%	**0.364**	0.342	**0.972**	1.075	**0.350**	0.171	**0.808**	0.291
	500	**96.1%**	6.6%	**0.474**	0.356	**1.431**	1.257	**0.388**	0.239	**1.532**	0.641
	750	**100.0%**	0.0%	**0.236**	0.287	**0.740**	1.085	**0.388**	0.230	**2.979**	0.602
	1000	**100.0%**	0.0%	**0.028**	0.023	**0.177**	0.004	**0.322**	0.007	**4.053**	0.065
**[C_4mim] [TFA]**	0	**0.0%**	0.0%	**0.000**	0.000	**0.000**	0.000	**0.000**	0.000	**0.000**	0.000
	125	**20.0%**	0.0%	**0.261**	0.253	**0.379**	0.348	**0.240**	0.219	**0.136**	0.093
	250	**33.5%**	27.5%	**0.536**	0.560	**1.107**	0.998	**0.188**	0.019	**1.035**	0.330
	500	**86.1%**	19.1%	**0.542**	0.610	**2.003**	2.198	**0.788**	0.049	**2.848**	1.160
	750	**76.2%**	33.6%	**0.456**	0.539	**1.094**	1.171	**0.357**	0.167	**2.336**	1.505
	1000	**100.0%**	0.0%	**0.429**	0.532	**0.920**	1.067	**0.583**	0.028	**3.950**	0.615
**[C_2mim] [Triflat]**	0	**0.0%**	0.0%	**0.000**	0.000	**0.000**	0.000	**0.000**	0.000	**0.000**	0.000
	50	**0.0%**	0.0%	**0.611**	0.525	**2.714**	2.327	**0.176**	0.132	**0.122**	0.083
	125	**33.3%**	0.0%	**0.588**	0.506	**2.701**	2.316	**0.367**	0.202	**0.234**	0.117
	250	**0.0%**	0.0%	**0.676**	0.579	**3.551**	3.015	**0.669**	0.390	**0.297**	0.172
	500	**26.7%**	26.7%	**0.462**	0.240	**1.875**	1.325	**0.176**	0.024	**0.603**	0.186
	750	**100.0%**	0.0%	**0.173**	0.009	**0.685**	0.556	**1.372**	0.173	**7.585**	0.387
	1000	**100.0%**	0.0%	**0.198**	0.070	**0.881**	0.742	**1.213**	0.367	**6.328**	1.867

Table S3: Dose-Dependent Mortality Rates of MRSA exposed to Ammonium based ILs and corresponding molecular response values for four selected spectral regions.

IL name	IL concentration [mM]	Mortality [%]		d-values for 2860-2845 cm^{-1}		d-values for 2935-2915 cm^{-1}		d-values for 1570-1515 cm^{-1}		d-values for 1690-1620 cm^{-1}	
		Mean	SD	Mean	SD	Mean	SD	Mean	SD	Mean	SD
[TMA][Cl]	0	**0.0%**	0.0%	**0.000**	0.000	**0.000**	0.000	**0.000**	0.000	**0.000**	0.000
	250	**4.3%**	25.9%	**0.444**	0.333	**1.404**	1.036	**0.186**	0.128	**0.106**	0.082
	500	**15.9%**	15.8%	**0.563**	0.396	**1.860**	1.169	**0.183**	0.125	**0.117**	0.089
	750	**12.5%**	12.5%	**0.973**	0.032	**3.168**	0.003	**0.277**	0.006	**0.166**	0.053
	1000	**8.8%**	12.4%	**0.685**	0.492	**2.245**	1.634	**0.220**	0.153	**0.155**	0.065
[TMC$_4$A][Cl]	0	**0.0%**	0.0%	**0.000**	0.000	**0.000**	0.000	**0.000**	0.000	**0.000**	0.000
	50	**0.0%**	0.0%	**0.560**	0.577	**1.826**	1.705	**0.329**	0.246	**0.073**	0.036
	125	**0.0%**	0.0%	**0.611**	0.629	**2.107**	2.022	**0.322**	0.241	**0.081**	0.050
	250	**-12.5%**	12.5%	**0.855**	0.008	**2.759**	0.173	**0.274**	0.201	**0.143**	0.053
	500	**12.5%**	12.5%	**1.397**	0.180	**4.427**	0.473	**0.256**	0.018	**0.156**	0.048
	750	**-25.0%**	25.0%	**1.513**	0.008	**4.696**	0.149	**0.247**	0.015	**0.226**	0.036
	1000	**62.5%**	0.0%	**1.382**	1.178	**4.348**	3.669	**0.252**	0.164	**0.393**	0.311
[TMC$_8$A][Cl]	0	**0.0%**	0.0%	**0.000**	0.000	**0.000**	0.000	**0.000**	0.000	**0.000**	0.000
	12.5	**91.2%**	7.6%	**0.464**	0.463	**1.340**	1.326	**0.176**	0.010	**0.929**	0.622
	25	**99.9%**	0.1%	**0.338**	0.337	**1.083**	1.061	**0.350**	0.028	**1.737**	0.679
	50	**100.0%**	0.0%	**0.299**	0.272	**0.910**	0.761	**0.495**	0.074	**2.549**	0.863
[TMC$_{10}$A][Cl]	0	**0.0%**	0.0%	**0.000**	0.000	**0.000**	0.000	**0.000**	0.000	**0.000**	0.000
	0.1	**0.0%**	0.0%	**0.685**	0.217	**2.019**	0.887	**0.241**	0.011	**0.148**	0.004
	0.5	**100.0%**	0.0%	**0.216**	0.105	**0.587**	0.254	**0.257**	0.004	**0.926**	0.009
	1	**99.6%**	0.6%	**0.268**	0.206	**0.683**	0.567	**0.301**	0.074	**1.601**	0.413
	5	**100.0%**	0.0%	**0.368**	0.246	**0.395**	0.191	**0.523**	0.103	**2.569**	1.002
[TMC$_{12}$A][Cl]	0	**0.0%**	0.0%	**0.000**	0.000	**0.000**	0.000	**0.000**	0.000	**0.000**	0.000
	0.1	**0.0%**	0.0%	**0.065**	0.063	**0.157**	0.137	**0.258**	0.197	**0.236**	0.161
	0.5	**0.0%**	0.0%	**0.769**	0.201	**1.943**	1.090	**0.220**	0.011	**0.338**	0.082
	1	**39.3%**	85.6%	**0.588**	0.190	**1.370**	0.545	**0.197**	0.043	**1.073**	0.330
	5	**100.0%**	0.0%	**0.273**	0.129	**0.554**	0.337	**0.320**	0.076	**2.685**	0.500
	10	**100.0%**	0.0%	**0.196**	0.164	**0.417**	0.374	**0.571**	0.091	**3.095**	0.540
[TMC$_{16}$A][Cl]	0	**0.0%**	0.0%	**0.000**	0.000	**0.000**	0.000	**0.000**	0.000	**0.000**	0.000
	0.05	**25.0%**	43.3%	**1.667**	0.522	**5.225**	1.763	**0.262**	0.035	**0.124**	0.049
	0.1	**25.0%**	43.3%	**1.662**	0.638	**5.184**	2.125	**0.218**	0.048	**0.193**	0.056
	0.5	**73.6%**	52.9%	**0.582**	0.367	**2.181**	1.138	**0.238**	0.081	**0.570**	0.223
	1	**91.7%**	16.5%	**0.409**	0.366	**1.582**	1.123	**0.281**	0.073	**0.682**	0.084
	5	**100.0%**	0.0%	**0.298**	0.232	**0.936**	0.424	**0.355**	0.134	**0.918**	0.106
[TC$_8$MA][Cl]	0	**0.0%**	0.0%	**0.000**	0.000	**0.000**	0.000	**0.000**	0.000	**0.000**	0.000
	0.01	**61.5%**	0.0%	**0.819**	0.736	**2.710**	2.398	**0.234**	0.184	**0.090**	0.070
	0.05	**80.7%**	19.6%	**0.128**	0.067	**0.478**	0.134	**0.180**	0.016	**0.432**	0.261
	0.1	**100.0%**	0.0%	**0.456**	0.246	**0.982**	0.620	**0.171**	0.003	**1.269**	0.373
	0.25	**100.0%**	0.0%	**3.305**	2.558	**5.460**	4.190	**0.265**	0.178	**2.471**	1.898
[DC$_8$DMA][Cl]	0	**0.0%**	0.0%	**0.000**	0.000	**0.000**	0.000	**0.000**	0.000	**0.000**	0.000
	0.05	**0.0%**	0.0%	**0.421**	0.122	**1.141**	0.449	**0.224**	0.053	**0.120**	0.041
	0.1	**0.0%**	0.0%	**0.301**	0.153	**0.892**	0.558	**0.193**	0.050	**0.120**	0.042
	0.5	**96.1%**	4.8%	**0.240**	0.230	**0.737**	0.699	**0.227**	0.035	**0.789**	0.288
	1	**100.0%**	0.0%	**0.343**	0.201	**0.965**	0.567	**0.299**	0.096	**1.230**	0.489

Table S4: Dose-Dependent Mortality Rates of EPEC exposed to [$C_n$mim][Cl] based ILs and corresponding molecular response values for four selected spectral regions.

IL name	IL concentration [mM]	Mortality [%] Mean	Mortality [%] SD	d-values for 2860-2845 cm^{-1} Mean	SD	d-values for 2935-2915 cm^{-1} Mean	SD	d-values for 1570-1515 cm^{-1} Mean	SD	d-values for 1690-1620 cm^{-1} Mean	SD
**[C_2mim] [Cl]**	0	**0.0%**	0.0%	**0.000**	0.000	**0.000**	0.000	**0.000**	0.000	**0.000**	0.000
	750	**53.8%**	28.8%	**0.025**	0.017	**0.065**	0.009	**0.172**	0.017	**0.085**	0.003
	1000	**88.2%**	5.6%	**0.032**	0.022	**0.051**	0.023	**0.100**	0.024	**0.106**	0.049
**[C_4mim] [Cl]**	0	**0.0%**	0.0%	**0.000**	0.000	**0.000**	0.000	**0.000**	0.000	**0.000**	0.000
	125	**25.8%**	0.0%	**0.034**	0.031	**0.043**	0.026	**0.191**	0.156	**0.089**	0.048
	250	**0.0%**	0.0%	**0.015**	0.031	**0.031**	0.017	**0.141**	0.116	**0.067**	0.037
	500	**18.0%**	18.0%	**0.024**	0.004	**0.032**	0.000	**0.134**	0.012	**0.081**	0.007
	750	**74.0%**	20.2%	**0.022**	0.008	**0.082**	0.044	**0.204**	0.109	**1.974**	3.244
	1000	**81.1%**	18.4%	**0.021**	0.018	**0.083**	0.046	**0.197**	0.133	**2.142**	3.513
**[C_6mim] [Cl]**	0	**0.0%**	0.0%	**0.000**	0.000	**0.000**	0.000	**0.000**	0.000	**0.000**	0.000
	50	**22.5%**	31.8%	**0.007**	0.004	**0.082**	0.019	**0.200**	0.060	**0.266**	0.092
	125	**67.7%**	40.1%	**0.015**	0.006	**0.061**	0.008	**0.183**	0.060	**0.635**	0.219
	250	**100.0%**	0.0%	**0.013**	0.003	**0.110**	0.018	**0.187**	0.053	**4.967**	2.287
	500	**100.0%**	0.0%	**0.014**	0.005	**0.150**	0.027	**0.275**	0.114	**6.308**	2.687
**[C_8mim] [Cl]**	0	**0.0%**	0.0%	**0.000**	0.000	**0.000**	0.000	**0.000**	0.000	**0.000**	0.000
	1	**0.0%**	0.0%	**0.061**	0.050	**0.184**	0.122	**0.188**	0.045	**0.102**	0.015
	5	**56.5%**	34.5%	**0.026**	0.010	**0.033**	0.016	**0.168**	0.061	**0.281**	0.172
	12.5	**93.7%**	6.5%	**0.043**	0.026	**0.055**	0.047	**0.143**	0.036	**1.058**	0.625
	25	**99.7%**	0.5%	**0.037**	0.016	**0.108**	0.052	**0.153**	0.041	**2.378**	0.756
	50	**100.0%**	0.0%	**0.025**	0.016	**0.151**	0.080	**0.139**	0.037	**2.937**	0.426
**[C_{10}mim] [Cl]**	0	**0.0%**	0.0%	**0.000**	0.000	**0.000**	0.000	**0.000**	0.000	**0.000**	0.000
	0.1	**22.6%**	17.6%	**0.027**	0.028	**0.074**	0.055	**0.060**	0.037	**0.039**	0.017
	0.5	**99.8%**	0.3%	**0.069**	0.030	**0.065**	0.009	**0.131**	0.043	**0.541**	0.048
	1	**99.8%**	0.3%	**0.041**	0.025	**0.201**	0.182	**0.112**	0.040	**0.780**	0.077
	5	**99.9%**	0.1%	**0.023**	0.009	**0.111**	0.032	**0.121**	0.039	**2.513**	0.241

Table S5: Dose-Dependent Mortality Rates of EPEC exposed to [C4mim][X] based ILs and corresponding molecular response values for four selected spectral regions.

IL name	IL concentration [mM]	Mortality [%]		d-values [a.u.] for 2860-2845 cm^{-1}		d-values for 2935-2915 cm^{-1}		d-values for 1570-1515 cm^{-1}		d-values for 1690-1620 cm^{-1}	
		Mean	SD	Mean	SD	Mean	SD	Mean	SD	Mean	SD
[C_4mim] [BF_4]	0	**0.0%**	0.0%	**0.000**	0.000	**0.000**	0.000	**0.000**	0.000	**0.000**	0.000
	10	**48.5%**	35.3%	**0.086**	0.055	**0.115**	0.070	**0.171**	0.077	**0.344**	0.189
	50	**98.1%**	1.9%	**0.126**	0.064	**0.112**	0.072	**0.083**	0.030	**1.427**	0.377
	125	**99.9%**	0.1%	**0.105**	0.046	**0.083**	0.031	**0.117**	0.051	**2.322**	0.533
	250	**100.0%**	0.0%	**0.057**	0.036	**0.051**	0.019	**0.132**	0.035	**3.021**	0.588
	500	**100.0%**	0.0%	**0.021**	0.020	**0.030**	0.025	**0.125**	0.102	**2.862**	2.412
[C_4mim] [HFB]	0	**0.0%**	0.0%	**0.000**	0.000	**0.000**	0.000	**0.000**	0.000	**0.000**	0.000
	50	**24.9%**	40.7%	**0.040**	0.016	**0.109**	0.033	**0.239**	0.031	**0.148**	0.054
	125	**86.9%**	9.3%	**0.084**	0.034	**0.089**	0.047	**0.235**	0.036	**0.273**	0.053
	250	**100.0%**	0.0%	**0.061**	0.035	**0.054**	0.015	**0.096**	0.023	**2.925**	0.286
	500	**100.0%**	0.0%	**0.013**	0.008	**1.045**	0.400	**0.326**	0.024	**9.595**	0.387
	750	**100.0%**	0.0%	**0.056**	0.048	**5.345**	4.468	**0.648**	0.538	**11.377**	9.610
[C_4mim] [PF_6]	0	**0.0%**	0.0%	**0.000**	0.000	**0.000**	0.000	**0.000**	0.000	**0.000**	0.000
	50	**0.0%**	0.0%	**0.128**	0.114	**0.103**	0.081	**0.369**	0.306	**0.133**	0.105
	125	**84.8%**	1.7%	**0.086**	0.026	**0.053**	0.019	**0.316**	0.055	**0.249**	0.019
	250	**98.7%**	1.8%	**0.101**	0.067	**0.133**	0.089	**0.352**	0.060	**0.666**	0.336
	500	**100.0%**	0.0%	**0.072**	0.029	**0.135**	0.086	**0.247**	0.073	**1.149**	0.712
	750	**100.0%**	0.0%	**0.085**	0.042	**0.069**	0.024	**0.105**	0.018	**2.397**	0.366
[C_4mim] [TFA]	0	**0.0%**	0.0%	**0.000**	0.000	**0.000**	0.000	**0.000**	0.000	**0.000**	0.000
	125	**28.6%**	28.6%	**0.060**	0.024	**0.146**	0.057	**0.155**	0.027	**0.225**	0.033
	250	**99.2%**	0.9%	**0.091**	0.058	**0.150**	0.093	**0.098**	0.031	**1.292**	0.232
	500	**100.0%**	0.0%	**0.075**	0.067	**0.125**	0.111	**0.135**	0.023	**2.318**	0.387
	750	**97.8%**	3.8%	**0.064**	0.055	**0.111**	0.105	**0.118**	0.064	**2.183**	1.177
	1000	**100.0%**	0.0%	**0.033**	0.027	**0.065**	0.028	**0.163**	0.003	**3.144**	0.350
[C_2mim] [Triflat]	0	**0.0%**	0.0%	**0.000**	0.000	**0.000**	0.000	**0.000**	0.000	**0.000**	0.000
	50	**0.0%**	0.0%	**0.063**	0.052	**0.242**	0.198	**0.244**	0.204	**0.084**	0.065
	125	**0.0%**	0.0%	**0.104**	0.087	**0.299**	0.240	**0.278**	0.234	**0.119**	0.094
	500	**57.6%**	42.2%	**0.059**	0.041	**0.097**	0.078	**0.237**	0.097	**2.057**	2.218
	750	**100.0%**	0.0%	**0.046**	0.001	**0.187**	0.152	**0.853**	0.171	**9.025**	0.037
	1000	**100.0%**	0.0%	**0.050**	0.033	**0.403**	0.308	**1.131**	0.443	**9.944**	1.088

Table S6: Dose-Dependent Mortality Rates of EPEC exposed to Ammonium based ILs and corresponding molecular response values for four selected spectral regions.

IL name	IL concentration [mM]	Mortality [%]		d-values for 2860-2845 cm^{-1}		d-values for 2935-2915 cm^{-1}		d-values for 1570-1515 cm^{-1}		d-values for 1690-1620 cm^{-1}	
		Mean	SD	Mean	SD	Mean	SD	Mean	SD	Mean	SD
[TMA][Cl]	0	**0.0%**	0.0%	**0.000**	0.000	**0.000**	0.000	**0.000**	0.000	**0.000**	0.000
	250	**43.0%**	39.1%	**0.082**	0.053	**0.200**	0.137	**0.201**	0.082	**0.151**	0.096
	500	**91.3%**	35.6%	**0.120**	0.099	**0.291**	0.275	**0.223**	0.084	**2.116**	2.471
	750	**99.7%**	0.4%	**0.181**	0.152	**0.482**	0.486	**0.218**	0.049	**3.425**	2.660
	1000	**100.0%**	0.0%	**0.586**	0.797	**1.832**	2.640	**0.185**	0.029	**3.323**	2.435
[TMC$_4$A] [Cl]	0	**0.0%**	0.0%	**0.000**	0.000	**0.000**	0.000	**0.000**	0.000	**0.000**	0.000
	50	**72.7%**	0.0%	**0.084**	0.074	**0.127**	0.107	**0.098**	0.080	**0.072**	0.056
	125	**72.7%**	0.0%	**0.065**	0.057	**0.089**	0.072	**0.109**	0.089	**0.119**	0.096
	250	**74.1%**	34.7%	**0.084**	0.051	**0.162**	0.105	**0.179**	0.014	**1.850**	2.271
	500	**66.7%**	47.1%	**0.072**	0.026	**0.125**	0.092	**0.179**	0.023	**2.505**	2.899
	750	**100.0%**	0.0%	**0.021**	0.016	**0.057**	0.025	**0.158**	0.021	**3.866**	2.731
	1000	**96.8%**	0.0%	**0.276**	0.237	**0.817**	0.670	**0.159**	0.131	**0.531**	0.446
[TMC$_8$A] [Cl]	0	**0.0%**	0.0%	**0.000**	0.000	**0.000**	0.000	**0.000**	0.000	**0.000**	0.000
	12.5	**0.0%**	81.9%	**0.018**	0.002	**0.044**	0.017	**0.193**	0.042	**0.272**	0.029
	25	**93.6%**	9.1%	**0.009**	0.003	**0.029**	0.001	**0.153**	0.034	**0.502**	0.167
	50	**100.0%**	0.0%	**0.032**	0.031	**0.092**	0.024	**0.184**	0.020	**1.551**	0.218
[TMC$_{10}$A] [Cl]	0	**0.0%**	0.0%	**0.000**	0.000	**0.000**	0.000	**0.000**	0.000	**0.000**	0.000
	0.1	**56.4%**	22.2%	**0.021**	0.006	**0.081**	0.017	**0.118**	0.017	**0.062**	0.014
	0.5	**98.9%**	1.2%	**0.025**	0.011	**0.056**	0.012	**0.085**	0.054	**0.384**	0.131
	1	**98.6%**	1.4%	**0.009**	0.003	**0.053**	0.013	**0.079**	0.046	**0.776**	0.186
	5	**100.0%**	0.0%	**0.228**	0.150	**0.294**	0.101	**0.077**	0.014	**1.648**	0.336
	10	**100.0%**	0.0%	**0.306**	0.053	**0.498**	0.101	**0.140**	0.001	**1.899**	0.661
	12.5	**100.0%**	0.0%	**0.343**	0.291	**0.486**	0.407	**0.066**	0.049	**2.230**	1.878
	25	**100.0%**	0.0%	**0.289**	0.244	**0.559**	0.462	**0.361**	0.265	**3.866**	3.198
[TMC$_{12}$A] [Cl]	0	**0.0%**	0.0%	**0.000**	0.000	**0.000**	0.000	**0.000**	0.000	**0.000**	0.000
	0.1	**-50.0%**	50.0%	**0.021**	0.009	**0.069**	0.018	**0.073**	0.063	**0.030**	0.019
	0.5	**48.9%**	23.9%	**0.020**	0.008	**0.051**	0.028	**0.153**	0.004	**0.140**	0.019
	1	**78.2%**	16.1%	**0.033**	0.018	**0.032**	0.009	**0.142**	0.023	**0.242**	0.104
	5	**99.9%**	0.2%	**0.010**	0.006	**0.058**	0.022	**0.074**	0.019	**1.248**	0.132
	10	**100.0%**	0.1%	**0.068**	0.075	**0.269**	0.182	**0.080**	0.015	**2.616**	0.817
[TMC$_{16}$A] [Cl]	0	**0.0%**	0.0%	**0.000**	0.000	**0.000**	0.000	**0.000**	0.000	**0.000**	0.000
	0.05	**8.3%**	31.2%	**0.097**	0.039	**0.415**	0.079	**0.117**	0.034	**0.054**	0.017
	0.1	**18.1%**	5.2%	**0.120**	0.059	**0.536**	0.313	**0.109**	0.039	**0.075**	0.034
	0.5	**70.0%**	60.0%	**0.076**	0.062	**0.612**	0.169	**0.087**	0.045	**0.490**	0.239
	1	**99.9%**	0.1%	**0.072**	0.078	**0.438**	0.079	**0.071**	0.025	**0.734**	0.208
	5	**100.0%**	0.0%	**0.062**	0.044	**1.054**	0.275	**0.217**	0.167	**0.969**	0.223
	10	**100.0%**	0.0%	**0.406**	0.347	**2.702**	1.382	**0.550**	0.153	**0.792**	0.067
	20	**100.0%**	0.0%	**1.369**	1.163	**5.958**	5.042	**0.810**	0.675	**0.675**	0.562
[TC$_8$MA] [Cl]	0	**0.0%**	0.0%	**0.000**	0.000	**0.000**	0.000	**0.000**	0.000	**0.000**	0.000
	0.01	**0.0%**	0.0%	**0.087**	0.077	**0.243**	0.196	**0.179**	0.149	**0.085**	0.068
	0.05	**66.1%**	46.8%	**0.050**	0.048	**0.439**	0.431	**0.131**	0.033	**0.254**	0.102
	0.1	**100.0%**	0.0%	**0.750**	0.574	**1.167**	0.367	**0.107**	0.006	**1.757**	1.492
	0.25	**100.0%**	0.0%	**0.789**	0.000	**1.507**	0.000	**0.032**	0.000	**0.663**	0.000
	0.5	**100.0%**	0.0%	**1.763**	1.150	**1.936**	0.888	**0.122**	0.063	**3.625**	2.776
	1	**100.0%**	0.0%	**4.479**	0.327	**3.556**	0.314	**0.258**	0.023	**6.761**	0.796
	2.5	**100.0%**	0.0%	**3.668**	3.102	**3.216**	2.713	**0.074**	0.058	**6.248**	5.277
[DC$_8$DMA] [Cl]	0	**0.0%**	0.0%	**0.000**	0.000	**0.000**	0.000	**0.000**	0.000	**0.000**	0.000
	0.05	**16.7%**	23.6%	**0.019**	0.013	**0.053**	0.034	**0.099**	0.035	**0.041**	0.008
	0.1	**0.0%**	0.0%	**0.025**	0.011	**0.091**	0.057	**0.079**	0.047	**0.056**	0.027
	0.5	**75.0%**	35.4%	**0.022**	0.004	**0.177**	0.068	**0.100**	0.069	**0.395**	0.267
	1	**99.8%**	0.2%	**0.048**	0.046	**0.263**	0.151	**0.103**	0.057	**0.596**	0.353
	1.25	**100.0%**	0.0%	**0.046**	0.041	**0.393**	0.321	**0.044**	0.032	**0.646**	0.543
	2.5	**100.0%**	0.0%	**0.137**	0.118	**0.459**	0.388	**0.058**	0.044	**1.375**	1.158
	5	**100.0%**	0.0%	**0.237**	0.206	**0.570**	0.482	**0.113**	0.087	**2.639**	2.227

Table S7: Peak shifts the asymmetric $\nu_{as}(CH_2)$ (near 2923 cm^{-1}) and symmetric $\nu_s(CH_2)$ (near 2852 cm^{-1}) stretching mode of CH_2 functional groups in fatty acids of MRSA at different IL concentrations.

	Concentration [mM]	$\nu_{as}(CH_2)$ Peak	$\nu_s(CH_2)$ Peak
**PBS**		2922	2852
**$[TMC_{16}A][Cl]$**	0.05	2920	2851
	0.1	2920	2851
	0.5	2922	2852
	1	2922	2852
	5	2922	2852
**$[TC_8MA][Cl]$**	0.01	2920	2851
	0.05	2923	2853
	0.1	2925	2852
	0.25	2925	2853
**$[TMC_{10}A][Cl]$**	0.1	2921	2852
	0.5	2922	2852
	1	2923	2852
	5	2924	2853
**$[C_4Mim][PF_6]$**	50	2920	2852
	125	2922	2852
	250	2922	2852
	500	2922	2852
	1000	2923	2852
